# Lifespan Extension by Retrotransposons under Conditions of Mild Stress Requires Genes Involved in tRNA Modifications and Nucleotide Metabolism

**DOI:** 10.3390/ijms251910593

**Published:** 2024-10-01

**Authors:** Patrick H. Maxwell, Mustafa Mahmood, Maya Villanueva, Kaitlyn Devine, Nina Avery

**Affiliations:** Biology Department, Siena College, Loudonville, NY 12211, USA

**Keywords:** retrotransposon, Ty1, chronological lifespan, aging, yeast, Saccharomyces

## Abstract

Retrotransposons are mobile DNA elements that are more active with increasing age and exacerbate aging phenotypes in multiple species. We previously reported an unexpected extension of chronological lifespan in the yeast, *Saccharomyces paradoxus*, due to the presence of Ty1 retrotransposons when cells were aged under conditions of mild stress. In this study, we tested a subset of genes identified by RNA-seq to be differentially expressed in *S. paradoxus* strains with a high-copy number of Ty1 retrotransposons compared with a strain with no retrotransposons and additional candidate genes for their contribution to lifespan extension when cells were exposed to a moderate dose of hydroxyurea (HU). Deletion of *ADE8*, *NCS2*, or *TRM9* prevented lifespan extension, while deletion of *CDD1*, *HAC1*, or *IRE1* partially prevented lifespan extension. Genes overexpressed in high-copy Ty1 strains did not typically have Ty1 insertions in their promoter regions. We found that silencing genomic copies of Ty1 prevented lifespan extension, while expression of Ty1 from a high-copy plasmid extended lifespan in medium with HU or synthetic medium. These results indicate that cells adapt to expression of retrotransposons by changing gene expression in a manner that can better prepare them to remain healthy under mild stress.

## 1. Introduction

A variety of factors have been found to contribute to functional decline during aging in many species, ranging from genomic instability and problems maintaining appropriate levels and functions of proteins (proteostasis) to mitochondrial dysfunction, changes in cell–cell communication, and exhaustion of stem cells [1]. Retrotransposons are transposable elements that are mobile through a copy–paste mechanism that involves the reverse transcription of an RNA intermediate [2]. Retrotransposon expression, mobility (retrotransposition), or both, have been shown to increase with age in a variety of organisms, including yeast, nematodes, fruit flies, mice, and human cells [3,4,5,6,7,8,9,10]. 

In some cases, retrotransposons and endogenous retroviruses have been shown to contribute to functional decline with aging. The fruit fly endogenous retrovirus *gypsy* contributes to neurodegeneration [11] and human *Alu* or mouse *Alu*-like B1 and B2 elements contribute to age-related macular degeneration [12]. Cytosolic reverse transcription of human *Alu* RNA causes retinal pigmented epithelium cell death via triggering an innate immune response to contribute to macular degeneration [13,14,15,16]. Cytosolic reverse transcription of human L1 retrotransposons triggers an interferon response in senescent cells [17]. Endogenous retroviruses are overexpressed with age in mice, primates, and humans [18]. Retroviral-like particles from these retroviruses can be transferred from old to young cells to trigger innate immune responses and senescence, and repressing those retroviruses diminishes the accumulation of senescent cells and loss of tissue function [18]. Virus-like particles (VLPs) formed by Ty retrotransposons in *Saccharomyces cerevisiae* contribute to protein aggregation, aggregate toxicity, and silencing Ty elements increased replicative lifespan in *S. cerevisiae* [19]. Despite all these negative impacts of retroelements on healthy aging, our group previously reported that the presence of Ty1 retrotransposons in the genome of the yeast, *S. paradoxus*, extended lifespan when cells were exposed to mild stress [20].

Ty1 elements are long-terminal repeat (LTR) retrotransposons abundant and active in many lab strains of *S. cerevisiae* [21]. Ty1 retrotransposition involves the translation of Ty1 mRNA into Gag and Gag-Pol fusion proteins, binding of Gag to Ty1 RNA, and assembly of Gag into VLPs that incorporate Ty1 RNA and Pol protein. Maturation of VLPs leads to the reverse transcription of Ty1 RNA via the Pol reverse transcriptase domain and ultimately integration of the resulting Ty1 cDNA at a genomic site via the Pol integrase domain [21]. We previously engineered a strain of *S. paradoxus* with no retrotransposons to have a low- (1-3) or high- (~20) copy number of Ty1 retrotransposons and compared their chronological lifespans [20]. Yeast chronological lifespan is determined by how long cells remain viable during stationary phase [22]. The high-copy Ty1 strains had longer chronological lifespans than the zero-copy parent strain under conditions of mild stress [20], including growth in rich medium containing a low dose of hydroxyurea (HU), which inhibits ribonucleotide reductase, or growth in synthetic medium that leads to acetic acid accumulation [23]. This retrotransposon-dependent lifespan extension was correlated with reduced reactive oxygen species during growth to stationary phase, but was not correlated with the level of Ty1 retrotransposition [20].

Here, we investigated yeast gene functions required for retrotransposon-dependent extension of chronological lifespan. We tested several candidate genes based on their relationship to Ty1 retrotransposition and aging. We also used RNA-seq to identify genes differentially expressed due to a high-copy number of Ty1 and a low dose of HU and identified >90 differentially expressed genes (DEGs) in high-copy Ty1 strains compared with the zero-copy Ty1 strain, most of which were overexpressed in high-copy strains. Several of these genes were required for full extension of lifespan by Ty1, including genes that contribute to tRNA modifications and nucleotide metabolism. Ty1 insertions were not generally found upstream of overexpressed genes, and expression of Ty1 from a plasmid extended lifespan. Overall, these results indicate that yeast cells are likely adapting to the expression of retrotransposons by changing gene expression in ways that lead to longer lifespan when they are stressed. 

## 2. Results and Discussion

### 2.1. Retrotransposons Extend Lifespan When Cells Are Exposed to Mild DNA Replication or DNA Damage Stress

**Figure 1:**
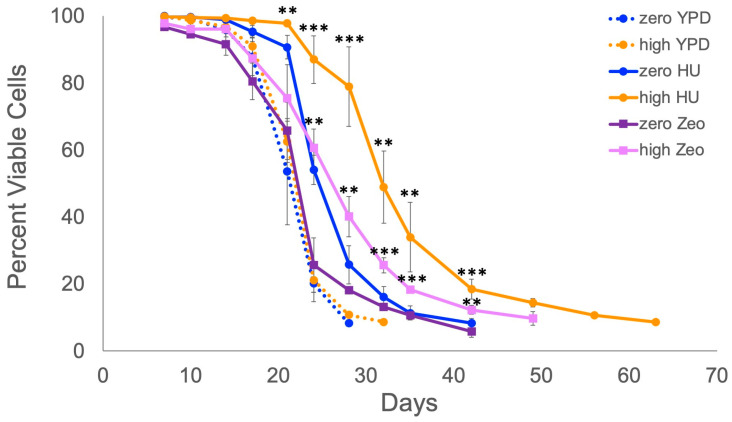
Ty1 extends chronological lifespan of yeast cells exposed to mild DNA damage or DNA replication stress. Viabilities of zero-copy (zero) or high-copy (high) Ty1 cell populations grown in control rich medium (YPD), YPD with 30 mM hydroxyurea (HU), or YPD with 1 µg/mL Zeocin (Zeo) were determined via trypan blue staining. Mean and standard deviation are shown for three (Zeo) or four trials (YPD, HU). Independent high-copy strains were used for each trial. Asterisks indicate significant differences for treated high-copy Ty1 strains compared with the correspondingly treated zero-copy strain: *p* < 0.01 (**) or *p* < 0.001 (***).

We previously reported that *S. paradoxus* strains with approximately 20 or more chromosomal copies of the Ty1 retrotransposon (high-copy Ty1 strains) have longer chronological lifespans than a Ty-less parent strain (zero-copy Ty1 strain) under mild stress conditions [20]. We confirmed that effect for one of those stresses, mild DNA replication stress, due to growth in medium containing 30 mM HU. The original zero-copy Ty1 strain and independent high-copy Ty1 derivatives were grown in regular YPD medium or YPD with HU, and cell viability was periodically determined until viability of the populations was below 10%. As we previously observed, treatment with a moderate dose of HU increased the chronological lifespan of the zero-copy Ty1 strain but increased the lifespans of the high-copy Ty1 strains to a greater extent (Figure 1). The days to reach 50% and 10% viability in HU medium increased by approximately 33% and 53% in high-copy Ty1 strains compared with the zero-copy Ty1 strain. Hereafter, we refer to days to reach 50% or 10% viability as median or maximum lifespan, respectively. We also measured chronological lifespans of cells grown in YPD with 1 µg/mL of the DNA-damaging agent Zeocin, a mild stress not tested in our prior study. This stress had only a small impact on the lifespan of the zero-copy Ty1 strain, but noticeably extended the lifespans of the high-copy Ty1 strains (Figure 1). The median and maximum lifespans increased by approximately 18% and 31% in treated high-copy Ty1 strains compared with the treated zero-copy Ty1 strain. These results show that lifespan extension by Ty1 retrotransposons is not specific to HU treatment, but likely is a more general response to mild DNA replication or damage stress. It is possible that a high-copy number of Ty1 retrotransposons could extend lifespan in the presence of other mild stresses, but we chose to further investigate how retrotransposons extend lifespan in the presence of HU for this study.

### 2.2. IRE1 and HAC1 Gene Functions Are Partly Required for Ty1-Dependent Lifespan Extension

We previously showed that the frequency of Ty1 retrotransposition is not correlated with lifespan extension, indicating that this effect is not simply due to the rate of ongoing retrotransposon insertions, and a low-copy number of Ty1 did not extend lifespan [20]. We therefore considered that high expression of Ty1 elements may impact certain cellular processes to contribute to lifespan extension. Ty1 is negatively regulated via autophagy [24], a process of degrading and recycling cellular components, and autophagy is known to contribute to lifespan extension resulting from mild stress in some contexts [25,26,27,28]. We tested whether autophagy is required for Ty1-dependent lifespan extension using *atg19∆* mutant strains lacking the Atg19p protein that interacts with Ty1 Gag protein [24], as well as *atg1∆* and *atg8∆* mutants that lack proteins required for autophagosome vesicle formation and membrane fusion [29,30,31].

**Figure 2:**
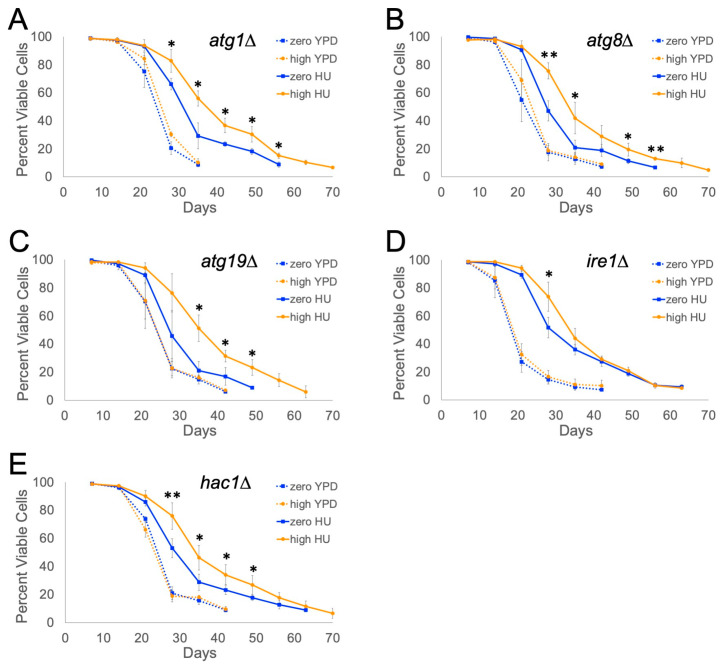
Contribution of autophagy and unfolded protein response genes to Ty1-dependent lifespan extension. Viabilities of (**A**) *atg1∆*, (**B**) *atg8∆*, (**C**) *atg19∆*, (**D**) *ire1∆*, or (**E**) *hac1∆* mutant zero- and high-copy Ty1 strains in control medium (YPD) or YPD with 30 mM HU (HU) were determined via trypan blue staining. Mean and standard deviation are shown for three trials (*atg* mutants) or four trials (*ire1∆* and *hac1∆*), using at least three independent high-copy Ty1 strains per experiment. Asterisks indicate significant differences for HU-treated high-copy strains compared with the HU-treated zero-copy strain: *p* < 0.05 (*) or *p* < 0.01 (**).

Defects in autophagy did not prevent Ty1 from extending lifespan in HU-treated cells, based on comparing zero-copy and high-copy Ty1 autophagy mutants (Figure 2A–C). In the absence of HU, these mutants had slightly longer maximum lifespans, as it took 30–40 days for the mutant populations to go below 10% viability, compared with not quite 30 days for the untreated wild-type cells shown in Figure 1. HU-treated high-copy Ty1 mutants maintained higher viabilities throughout the experiments compared with the HU-treated zero-copy mutants, though the magnitude of lifespan extension was slightly less than for the wild-type strains (compare Figure 1 to Figure 2A–C). The HU-treated high-copy Ty1 *atg1∆*, *atg8∆*, or *atg19∆* mutants had median lifespans 19%, 20%, or 31% longer than the corresponding HU-treated zero-copy mutants, and their maximum lifespans increased by 16%, 25%, or 25%, respectively. Overall, these data indicate that autophagy is not required for lifespan extension by Ty1 but may modestly contribute to the magnitude of the effect.

Prior work has shown that Ty1 Gag is translocated into the endoplasmic reticulum (ER) during translation [32], so we considered whether a high-copy number of Ty1 could trigger mild ER stress and the unfolded protein response (UPR). In baker’s yeast, the UPR is mediated by the Ire1p ER transmembrane protein that acts as an endoribonuclease to initiate splicing of *HAC1* mRNA when activated due to increased levels of unfolded proteins in the ER [33,34]. This allows translation of Hac1p for expression of genes involved in the UPR [35]. We individually deleted the *IRE1* or *HAC1* genes to determine if they were required for lifespan extension, which would implicate the UPR in lifespan extension. Chronological lifespans of the zero- and high-copy Ty1 *ire1∆* or *hac1∆* mutants were similar to those observed for the *atg* mutants in the control medium (Figure 2). The lifespan curve for HU-treated zero-copy *ire1∆* mutants was nearly the same as the HU-treated high-copy mutants (Figure 2D). Median lifespan was still extended by 16% in the high-copy Ty1 *ire1∆* mutants, but there were no differences in viabilities during the last half of the trials. The *hac1∆* zero- and high-copy Ty1 mutants also did not have any difference in their maximum lifespans, but the high-copy mutants maintained higher viabilities over nearly the whole course of the trials (Figure 2E). Median lifespan was extended in the HU-treated high-copy *hac1∆* mutants by 17% compared with the zero-copy mutants, similar to what was observed for the *ire1∆* mutants.

**Figure 3:**
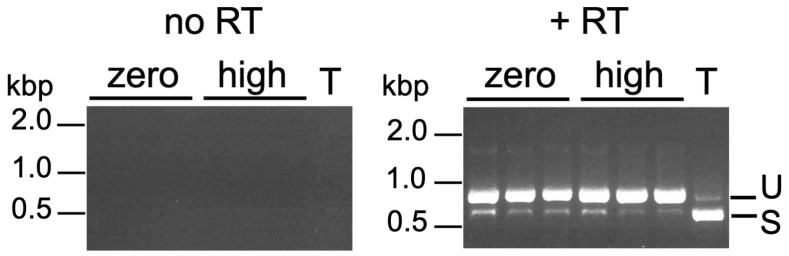
*HAC1* splicing was not induced by the presence of Ty1 in HU-treated cells. RT-PCR was performed using total RNA from three biological replicates of the zero-copy Ty1 strain (zero) or three different high-copy Ty1 strains (high) grown in YPD with 30 mM HU. As a positive control, the zero-copy strain was also treated with 1 µg/mL tunicamycin (T). The left and right images show control reactions without reverse transcriptase (no RT) and the experimental reactions (+ RT), respectively. The primers were predicted to amplify an 896 or 647 bp product from unspliced (U) or spliced (S) *HAC1* mRNA, respectively.

We directly tested whether the presence of Ty1 could induce the UPR through testing for *HAC1* splicing. The *HAC1* gene in the reference *S. paradoxus* genome is not annotated with an intron, but we determined the likely site of the intron by aligning the *S. paradoxus* sequence, including 272 base pairs downstream of the annotated stop codon (positions 95,579–96,537 in GenBank accession NC_047492), to the *S. cerevisiae HAC1* sequence (positions 75,179–76,147 in GenBank accession NC_001138) using the EMBOSS Needle pairwise sequence alignment tool [36] (Figure S1). Zero- and high-copy Ty1 strains were grown in YPD with HU to late exponential phase, total RNA was extracted, cDNA was synthesized, and *HAC1* sequences were amplified using PCR primers flanking the intron site. There was virtually no splicing of *HAC1* mRNA in the zero- or high-copy Ty1 strains, but most of the *HAC1* mRNA was spliced when the zero-copy strain was treated with tunicamycin, a known inducer of the UPR (Figure 3). 

Overall, these data indicate that Ty1 is not stimulating the UPR to cause lifespan extension, but deletion of *IRE1* or *HAC1* prevent Ty1 from increasing maximum lifespan. This could reflect a partial requirement for the UPR for Ty1 to increase longevity. Recent work has shown that Ty1 expression and VLP formation contribute to localization of protein aggregates at mitochondria, increased protein aggregation overall, and increased toxicity due to expression of a misfolding variant of a Huntingtin exon 1 protein [19]. Considering these negative impacts of Ty1 on proteostasis, perhaps the UPR contributes to overall protein quality control in the context of Ty1 expression to allow for full lifespan extension. However, the greater impact of deleting *IRE1* raises the possibility that functions of *IRE1* not related to the splicing of *HAC1* could contribute to lifespan extension, such as its role in inositol metabolism [37]. An alternative possibility is that deletion of *IRE1* or *HAC1* creates a mild stress through impairing the UPR that interacts with the mild stress caused by HU to increase lifespan, and that the presence of Ty1 has only a modest impact on the interaction of those stresses. 

### 2.3. Differential Gene Expression in the Presence of Ty1 and HU

**Figure 4:**
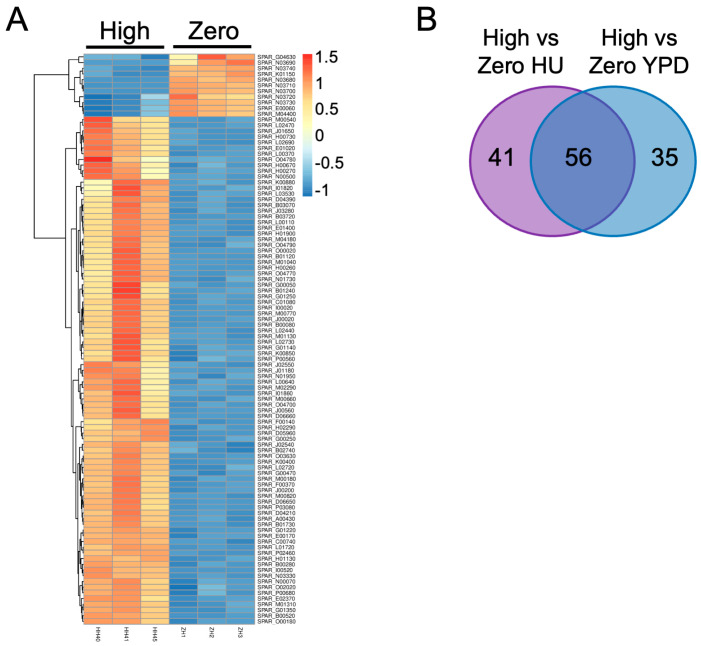
Genes differentially expressed due to the presence of Ty1 and HU. (**A**) Heat map of genes underexpressed (blue) or overexpressed (red) in the high- versus zero-copy Ty1 strains in YPD with HU. Note that two genes downregulated in the high-copy strains, *SPAR_K01150* and *SPAR_G04630*, are present in the heat map, but are not discussed in the main text as downregulated because their changes were less than two-fold. The color scale to the right indicates log_2_ fold-changes. (**B**) Venn diagram of differentially expressed genes common and specific to each media condition.

We carried out RNA-seq analysis to explore gene functions contributing to the positive influence of Ty1 on chronological lifespan in a less biased manner than our candidate gene analysis. Three biological replicates of the zero-copy Ty1 strain and three independent high-copy Ty1 strains (SPA40, SPA41, and SPA45) were grown to stationary phase in YPD or YPD with HU, and RNA extracts were prepared for poly(A) RNA sequencing. Analyzing these samples allowed us to avoid focusing on genes differentially expressed in only one high-copy Ty1 strain and to identify genes differentially expressed due to the combination of Ty1 and HU. A total of 5428–5448 mRNAs were identified in each of the 12 samples. Comparing the high-copy Ty1 strains with the zero-copy Ty1 replicates in YPD with HU identified 97 genes differentially expressed in all three high-copy Ty1 strains by at least two-fold (Figure 4A). Most differentially expressed genes (DEGs) were overexpressed in the high-copy Ty1 strains treated with HU, with only nine downregulated genes. The same comparison for strains grown in YPD without HU identified 91 DEGs, and 56 DEGs were common to both media conditions, including all the downregulated genes in HU medium (Figure 4B). The full list of DEGs specific or common to the two media conditions are provided as Supplementary Material (Tables S1–S3).

To investigate cellular functions/processes or pathways that may be contributing to lifespan extension, gene set enrichment analysis (GSEA) was performed with GO terms and KEGG pathways gene sets using RNA-seq data from the HU or YPD-only datasets (Table 1 or Table 2, respectively). Several GO terms were enriched for both media conditions and are indicated in bold (Table 1). Five diverse GO terms were enriched specifically in the HU dataset, related to iron homeostasis, glutamine metabolism, tRNA metabolism, stress responses, and microtubule nucleation. More GO terms were enriched in the YPD-only dataset (Table 1). In particular, eight terms related to ribosomes and translation were enriched. Interestingly, multiple genes known to facilitate Ty1 retrotransposition encode ribosomal proteins and ribosome assembly factors. Individual deletions of those genes affect levels of Ty1 Gag protein, translational frameshifting required to produce Ty1 Pol proteins, and increase the levels of a truncated Ty1 Gag protein (p22) that decreases retrotransposition [38,39,40]. The GSEA results raise the possibility that expression of Ty1 changes the regulation of ribosomes and translation, which could indicate a more intimate connection between ribosome regulation and retrotransposons. These results could also reflect the recently identified role of Ty1 VLPs in affecting the extent and localization of protein aggregates [19], as overall changes in proteostasis could also influence expression of ribosomal proteins and assembly factors.

The GSEA of the pathways identified more enriched pathways for the HU dataset than the YPD-only dataset, with just two pathways in common (Table 2). Three pathways in the HU dataset related to amino acid metabolism and two to coenzyme metabolism (thiamine and pantothenate/CoA, Table 2), indicating that central aspects of metabolism could be involved in lifespan extension. The last HU-specific pathway was mismatch repair, which could potentially be beneficial for aging in the presence of mild DNA replication stress. A different DNA repair pathway was enriched for the YPD-only dataset (Table 2). Pyrimidine metabolism was enriched in both datasets and changes in regulation of nucleotide metabolism could potentially be beneficial for cells experiencing mild depletion of deoxynucleotides due to HU treatment. Overall, we expected GO terms and KEGG pathways specifically enriched in the presence of both Ty1 and HU to be better candidates for contributing to lifespan extension. However, it is possible that terms or pathways enriched in high-copy Ty1 strains in both media conditions could be contributing to lifespan extension, but do not result in a beneficial phenotype until cells are exposed to stress.

Considering that diverse processes and pathways were potentially implicated in Ty1-dependent lifespan extension through the GSEA, we also evaluated genes overexpressed by at least three-fold in high-copy Ty1 strains treated with HU to identify genes potentially contributing to lifespan extension. Twenty-four genes with diverse functions met these criteria (Table 3). The most highly overexpressed gene, *POP3*, encodes a subunit of RNase MRP, which cleaves pre-rRNA molecules, and RNase P, which cleaves tRNA precursors [41]. We chose not to further examine the role of *POP3* in lifespan extension in this study, though, because it is an essential gene that cannot be deleted. *NCS2* and *TRM9* are the next most highly overexpressed genes, and both encode proteins that contribute to tRNA modifications. A third gene on the list, *NCS6*, also encodes a tRNA-modifying enzyme (Table 3). These three genes seemed like promising candidates for contributing to lifespan extension, especially considering enrichment for the GO term tRNA modification in the presence of Ty1 and HU (Table 1). Four genes encode enzymes that contribute to nucleotide metabolism: *CDD1*, *FOL3*, *ADE8*, and *PRS1* (Table 3). Cdd1p converts cytidine to uridine [42], Fol3p catalyzes production of dihydrofolate that can be used for nucleotide synthesis [43], Ade8p catalyzes a step in de novo purine nucleotide synthesis [44], and Prs1p forms phosphoribosyl pyrophosphate than can be used for nucleotide synthesis [45]. Combined with the enrichment for pyrimidine metabolism in the presence of Ty1 (Table 2) and knowing that HU creates DNA replication stress, we chose to also further investigate the roles of some of these nucleotide metabolism genes. 

### 2.4. Genes Overexpressed in High-Copy Ty1 Strains Treated with HU Contribute to Lifespan Extension

**Figure 5:**
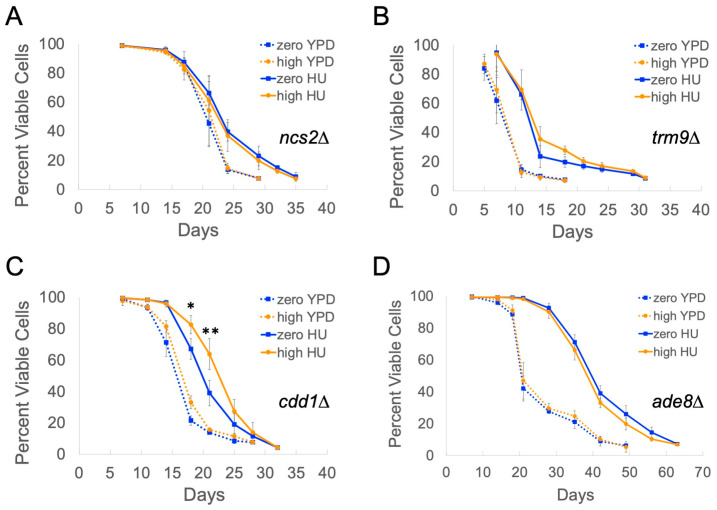
Genes involved in tRNA modifications and nucleotide metabolism contribute to Ty1-dependent lifespan extension. Mean and standard deviation for viabilities determined via trypan blue staining are shown for four trials each for (**A**) *ncs2∆*, (**B**) *trm9∆*, (**C**) *cdd1∆*, or (**D**) *ade8∆* mutants in control medium (YPD) or with 30 mM HU added (HU). At least three independent high-copy Ty1 strains were used for each experiment. Asterisks indicate significant differences for HU-treated high-copy Ty1 mutants compared with HU-treated zero-copy mutants: *p* < 0.05 (*) or *p* < 0.01 (**).

We measured the chronological lifespans of the zero-copy strain and the SPA40, SPA41, and SPA45 high-copy Ty1 strains used for RNA-seq individually deleted for *NCS2*, *TRM9*, *CDD1*, or *ADE8* in YPD and YPD with HU to determine whether any deletions affected lifespan in general, in addition to affecting Ty1-dependent lifespan extension. The *trm9∆* mutants had substantially shorter lifespans and *ade8∆* mutants had longer lifespans than the wild-type strains in control medium (compare Figure 5B,D to Figure 1). Lifespan extension by Ty1 was fully blocked in *ncs2∆*, *trm9∆*, and *ade8∆* mutants, and partially blocked in *cdd1∆* mutants (Figure 5). There was still a 14% increase in median lifespan in high-copy Ty1 *cdd1∆* mutants treated with HU compared with the zero-copy strain, but Ty1 was not able to extend maximum lifespan in any of the four mutants. These results confirm that at least some of the genes overexpressed in HU-treated high-copy Ty1 strains are contributing to lifespan extension.

The shorter lifespans of the *trm9∆* mutants in both media conditions could result from the contribution of Trm9p tRNA methyltransferase activity to the translation of a variety of proteins encoded by mRNAs enriched for AGA and GAA codons [46]. Loss of yeast *TRM9* leads to lower levels of ribosomal proteins and proteins involved in the DNA damage response, cell cycle control, protein translation, and protein folding [46,47]. Furthermore, loss of *TRM9* leads to increased rates of misincorporating amino acids during translation that result in greater levels of protein aggregation [48]. Expression of Ty1 proteins to form VLPs increases protein aggregation and toxicity of aggregates [19]. One possible interpretation of these results is that a modest impact of Ty1 on protein aggregation leads to *TRM9* overexpression and longer lifespans in HU-treated cells. The loss of *TRM9* could lead to a more severe negative impact on proteostasis that prevents the effect of Ty1 from being beneficial. As previously noted, ribosomal proteins and biogenesis factors facilitate Ty1 expression and retrotransposition [38,39,40]. Ty1 VLP maturation and retrotransposition are also regulated via the DNA damage response and DNA repair proteins [49,50,51]. An alternative interpretation is that loss of *TRM9* may interfere with interactions between Ty1 and protein translation or the DNA damage response, and thereby prevent Ty1 from increasing chronological lifespan when cells are exposed to mild stress.

Loss of *NCS2* prevents 2-thiolation of the wobble uridine position of specific tRNAs and has been shown to result in ribosomal pausing at specific codons and increased protein aggregation [52]. Clearance of protein aggregates induced by stress was slower in mutants lacking both *NCS2* and a second gene involved in modifying the wobble uridine position [52]. Ncs6p was also overexpressed in high-copy Ty1 strains treated with HU (Table 3) and works together with Ncs2p to form 2-thiouridine at the wobble uridine position [53]. Considering the observations that Ty1 expression and loss of Trm9p or Ncs2p activity all affect protein aggregation, we suggest that lifespan extension by Ty1 could involve changes in protein translation and aggregation that increase expression of tRNA-modifying enzymes to maintain proteostasis. 

Overexpression of genes involved in nucleotide salvage and synthesis pathways, such as *CDD1* and *ADE8*, could potentially be beneficial for cells experiencing DNA replication stress. Prior work has shown that Ty1 expression is increased due to severe adenine starvation in adenine-deprived cells when the Bas1p transcriptional activator of *ADE* genes is also absent [54], but it is not clear whether aging in the absence of *CDD1* or *ADE8* could create a similar severe condition to change Ty1 expression. Changes in nucleotide levels due to these gene deletions and nutrient depletion during chronological aging could potentially affect multiple other pathways, though, so the effect of these genes may not be direct. For instance, purine starvation can induce expression of the Gcn4p transcriptional activator that regulates hundreds of genes [55,56]. Loss of *ADE8* combined with purine starvation leads to increased levels of storage carbohydrates, such as trehalose, and increased resistance to stresses, including heat, oxidative damage, and desiccation [57]. Changes in stress tolerance in *ade8∆* mutants could potentially affect the sensitivity of cells to the HU treatment and interfere with the ability of Ty1 expression to have a positive effect on lifespan.

### 2.5. Ty1 Expression Causes Lifespan Extension

**Figure 6:**
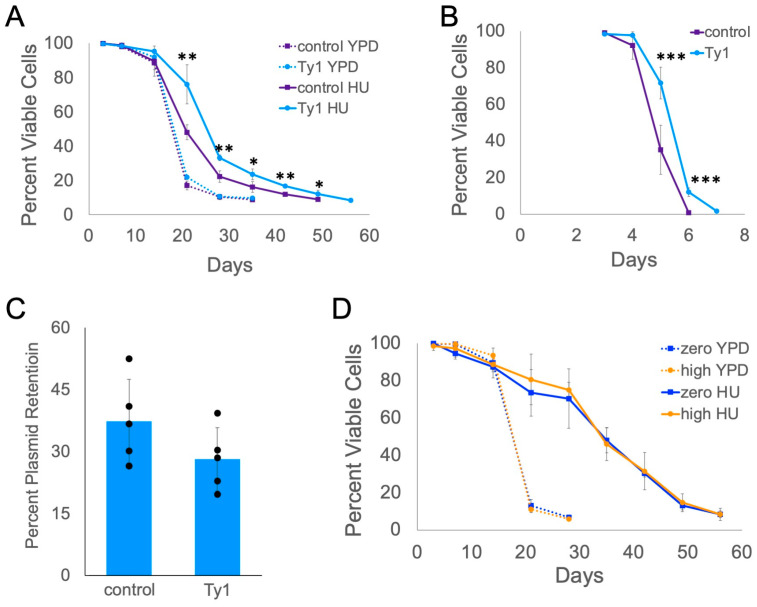
Expression of Ty1 results in lifespan extension. Derivatives of the zero-copy strain with a control plasmid expressing *kanMX* (control) or pTy1H3*kanMX* (Ty1) were aged in (**A**) YPD with G418 (YPD) or YPD with G418 and HU (HU), or (**B**) SC-ura broth. (**C**) The fraction of Ura^+^ cells after three days of growth of the zero-copy strain with each plasmid is expressed as a percentage. (**D**) *spt3∆* derivatives of the zero-copy strain (zero) and three different high-copy Ty1 strains (high) were aged in YPD without or with 30 mM HU. Viabilities were determined via trypan blue staining. Mean and standard deviation are shown for four (**A**), five (**B**,**C**), or three (**D**) trials. Asterisks indicate significant differences between control and Ty1-expressing cells in HU (**A**) or SC-ura medium (**B**): *p* < 0.05 (*), *p* < 0.01 (**), *p* < 0.001 (***).

Based on the prior discussions, we considered it likely that expression of Ty1 is causing gene expression changes that contribute to longer lifespan. Our previous study did not fully establish whether expression of Ty1, rather than its insertion pattern in the genome and effect on neighboring genes, is the cause of lifespan extension [20]. We introduced the high-copy pTy1H3*kanMX* plasmid to express a Ty1 element from its native promoter with the *kanMX* antibiotic-resistance gene downstream of Ty1 coding sequences into the zero-copy Ty1 strain. Zero-copy strains with pTy1H3*kanMX* had the same lifespan as zero-copy strains with a control plasmid only expressing *kanMX* in YPD with G418 antibiotic (Figure 6A). In contrast, strains with pTy1H3*kanMX* had longer lifespans than strains with the control plasmid in YPD with G418 and HU (Figure 6A). Median lifespan was extended by 22%, but maximum lifespan was only extended by 13%. We previously reported that chronological lifespan of the zero-copy strain was very short in SC medium, which could be due to the accumulation of acetic acid when cells age in SC [23], but high-copy Ty1 strains had moderately longer chronological lifespans (20–25%) in SC medium [20]. We repeated the lifespan experiments using SC medium lacking uracil (SC-ura) to select for the *URA3* gene present in each plasmid. Again, the cells with pTy1H3*kanMX* had longer lifespans than those with the control plasmid (Figure 6B). Median lifespan was extended by 15%, but maximum lifespan was only extended by 7%. 

Plasmid expression of Ty1 had more substantial effects on median than maximum lifespan, and we wondered whether this could result from some cells losing the plasmid during stationary phase. We grew cells with the control plasmid or pTy1H3*kanMX* in SC-ura to late log/early stationary phase and determined the percentage of cells that were still Ura^+^ prototrophs due to retention of the plasmid. On average, less than 40% of cells still had the respective plasmid (Figure 6C). This indicates that the effect of Ty1 expression on lifespan is likely diminished in these populations because not all the stationary phase cells are still expressing Ty1. 

To further explore the requirement for Ty1 expression, we determined the lifespans of zero- and high-copy Ty1 strains with a deletion of *SPT3*. Loss of *SPT3* function strongly reduces transcription of Ty1 elements and is commonly used as a mutation to block expression and retrotransposition of chromosomal Ty1 elements [58,59]. We observed no difference in the lifespans of zero- and high-copy Ty1 *spt3∆* mutants both in YPD and YPD with HU (Figure 6D), indicating that Ty1 can no longer extend lifespan in HU medium when it is not being expressed. The *spt3∆* mutants lived longer than the wild-type zero-copy strain in YPD with HU (compare Figure 1 and Figure 6D). This could result from Spt3p being a member of the SAGA (Spt-Ada-Gcn5 acetyltransferase) transcriptional control complex that is known to contribute to expression of genes induced by stress [60,61]. Loss of Spt3p could result in changes in stress responses that cause the HU-treatment to have a slightly more positive effect on lifespan. 

Overall, these results support that Ty1 expression causes cells to change their patterns of gene expression in a manner that can extend their lifespan in certain stress conditions. In particular, the results of expressing Ty1 from a plasmid indicate that changes in expression of genes nearby chromosomal Ty1 insertions are not required to extend lifespan. Combined with the lack of correlation between levels of retrotransposition and lifespan extension [20], it is likely that an intermediate step of Ty1 replication triggers cellular changes that can promote longevity in some contexts, such as expression of Ty1 proteins, association of Ty1 proteins and RNA, or formation of VLPs. Ty1 VLPs appear to interact with factors involved in protein quality control, and their formation can increase protein aggregation and aggregate toxicity [19]. Changes in proteostasis due to Ty1 expression could be one way that Ty1 expression is extending lifespan with mild stress, which is consistent with our speculation on the possible role of tRNA-modifying genes in lifespan extension (Section 2.4). 

### 2.6. Ty1 Insertions Are Not Typically Nearby Promoters of Overexpressed Genes

Based on our prior data that different high-copy Ty1 strains have distinct patterns of Ty1 insertions [20] and the data in Figure 6, we considered it unlikely that the gene expression changes we observed in high-copy strains directly resulted from insertions of Ty1 in or nearby those DEGs. The great majority of gene expression changes both in the YPD-only and HU datasets were due to overexpression (Section 2.3, Tables S1–S3). If these changes were frequently caused by Ty1 insertions, we would expect that the insertions would have needed to be present in or nearby the promoters of those genes to cause their overexpression. 

**Figure 7:**
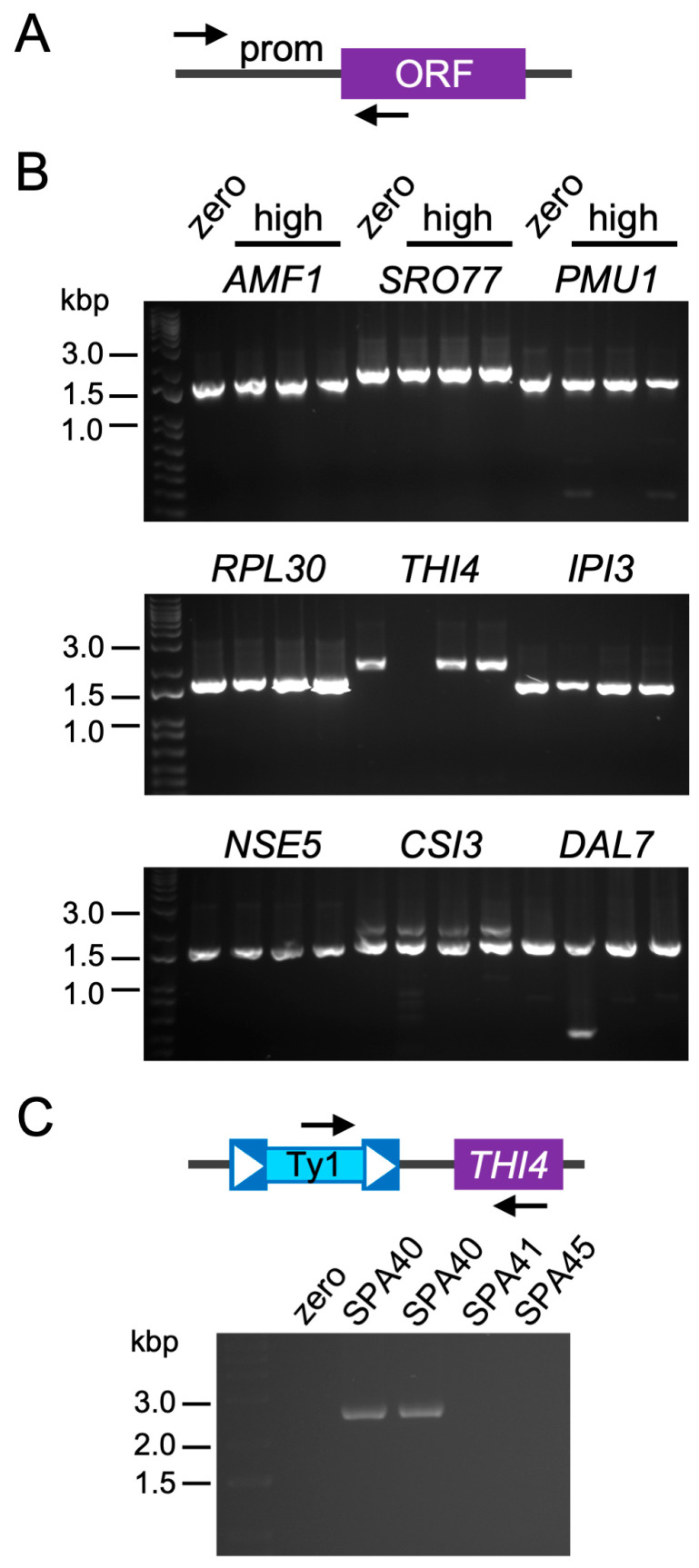
Ty1 is not typically inserted upstream of overexpressed genes. (**A**) Diagram of relative annealing positions of PCR primers (small arrows) upstream of promoter sequences (prom) and within open reading frames (ORF). (**B**) Example PCR products from the zero-copy strain (zero) and the three different high-copy Ty1 strains used for RNA-seq (high) for overexpressed genes in the YPD with HU dataset (top panel), the YPD-only dataset (middle panel), or common to both datasets (bottom panel) analyzed by agarose gel electrophoresis. The relevant gene names are shown above each set of four lanes. The leftmost lane in each image is a DNA ladder, and positions of selected marker bands in kilobase pairs (kbp) are indicated to the left of the images. (**C**) Diagram of relative positions of primers to detect Ty1 upstream of the *THI4* gene in the SPA40 high-copy Ty1 strain and agarose gel electrophoresis of PCR products. SPA40, SPA41, and SPA45 are the three independent high-copy Ty1 strains used for RNA-seq, and duplicate templates were tested for SPA40.

We designed PCR primers to amplify from coding sequences to about 1 kbp upstream of the ten most overexpressed genes specific to the HU or YPD-only datasets, and the six most overexpressed genes common to both datasets (Figure 7A, Tables S1–S3). These primers were used with templates prepared from the zero-copy strain and all three independent high-copy Ty1 strains used for RNA-seq (SPA40, SPA41, and SPA45). We successfully amplified upstream sequences from all 26 genes from the zero-copy and all three high-copy Ty1 strains used for RNA-seq, except for one gene (*THI4*) in one high-copy strain (Figure 7B). In that one case, Ty1 was present ~1 kbp upstream of the coding region with the 3′ LTR of Ty1 towards the gene (Figure 7C). The PCR results confirm that Ty1 insertions near or in promoters were not generally responsible for genes being overexpressed in the high-copy strains. Overexpression of these genes is therefore likely a response to the expression of Ty1 elements in the high-copy strains.

**Figure 8:**
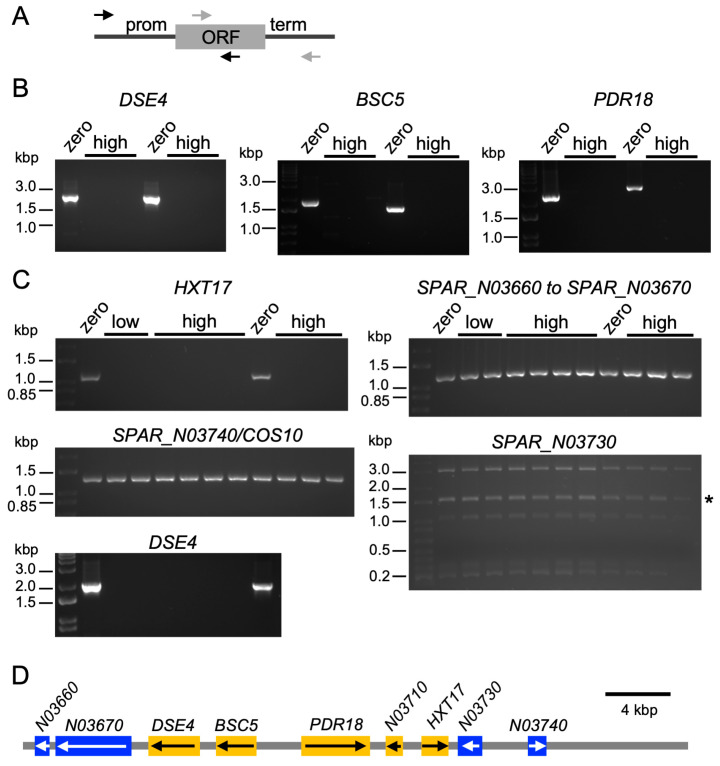
Some genes downregulated in high-copy Ty1 strains in both media conditions are absent from the high-copy strains. (**A**) Diagram showing relative positions of primer pairs (small arrows) to detect coding (ORF) and flanking sequences of downregulated genes. (**B**) Agarose gel electrophoresis of example PCR results for downregulated genes in the zero-copy Ty1 (zero) and three high-copy Ty1 (high) strains. The first lane in each image is a DNA ladder, and positions of selected marker bands are indicated in kilobase pairs (kbp). (**C**) Example PCR results using additional high-copy Ty1 and two low-copy Ty1 (low) strains testing for coding and flanking sequences of other genes near the right-arm telomere of Chr XIV. Asterisk indicates predicted product for *SPAR_N03730*. (**D**) Scale diagram of the terminal ~40 kbp of the right arm of Chr XIV for *S. paradoxus* genome assembly ASM207905v1, indicating open reading frames present (blue) or absent (orange) from the Ty1-containing strains. Arrows indicate orientations of open reading frames. Bar indicates scale.

We also designed primers to test for coding and flanking sequences of four genes downregulated by >100-fold in high-copy Ty1 strains in both media conditions: *DSE4*, *BSC5*, *PDR18*, and *SPAR_N03710* (Tables S3 and S4, Figure 8A). We unexpectedly failed to amplify all four genes from all three high-copy strains (Figure 8B). These genes are adjacent to each other near the telomere of the right arm of ChrXIV in the *S. paradoxus* reference sequence (assembly ASM207905v1). We designed primers to additional genes surrounding these four genes, including *SPAR_N03660*, *SPAR_N03670*, *SPAR_N03720/HXT17*, *SPAR_N03730*, and *SPAR_N03740/COS10*. The latter three genes are downregulated in high-copy Ty1 strains in both media conditions (Table S3). We also tested additional high-copy strains and two low-copy Ty1 strains. No PCR products were obtained for *DSE4*, *BSC5*, *PDR18*, *SPAR_N03710*, or *HXT17* from any of the low- or high-copy Ty1 strains we tested (Figure 8C). 

The absence of five genes adjacent to each other on Chr XIV (Figure 8D) in strains with low- and high-copy numbers of Ty1 indicates that a chromosomal deletion/rearrangement that deleted these five genes likely occurred during subculturing of the zero-copy strain in preparation for introducing Ty1 elements into the strain. There were variable levels of transcripts for these five genes in the zero-copy strain, with high expression of *SPAR_N03710*, but low to moderate expression of the other four genes (Table S3). The PCR results (Figure 8) indicate that the downregulation of those five genes in the high-copy Ty1 strains (Table S3) is not due to the presence of Ty1, though, but rather to loss of that genomic region. Low-copy Ty1 strains are also missing these five gene sequences (Figure 8B,C) but do not exhibit lifespan extension under conditions of mild stress [20], so the loss of these five genes does not likely contribute to the lifespan extension observed in the high-copy Ty1 strains. 

Overall, the data in Figure 6 support that Ty1 expression is responsible for lifespan extension, and the PCR results show that Ty1 is not typically inserted near promoters of genes to drive their overexpression in high-copy Ty1 strains (Figure 7). This indicates that the gene expression changes in the RNA-seq data are likely a response of the yeast cells to the expression of Ty1. At least some of those changes in gene expression are contributing to Ty1-dependent lifespan extension when cells are exposed to mild stress. 

## 3. Materials and Methods

### 3.1. Yeast Strains and Media

Yeast strains were grown using YPD medium (2% peptone, 1% yeast extract, and 2% glucose (*w*/*v*)) and standard synthetic medium (SC, 0.67% yeast nitrogen base without amino acids, 0.07% complete supplement mix (Sunrise Science Products, Knoxville, TN, USA), and 2% glucose (*w*/*v*)) with 2% agar added for solid medium [62]. All strains were derived from a Ty-less *S. paradoxus* strain, DG1768 (*MATalpha, his3-∆200hisG, ura3*, kindly provided by David Garfinkel, University of Georgia, Athens, GA, USA) [63]. Derivatives with approximately 20 or more copies of Ty1 integrated into their genomes were described previously and are referred to as high-copy Ty1 strains [20], in contrast to the DG1768 zero-copy Ty1 parent strain. The zero-copy strain and independent high-copy Ty1 strains with single gene deletions were made by lithium-acetate transformation of PCR products of *S. cerevisiae HIS3* with homology to sequences flanking the target gene at either end to replace the target gene coding sequences with the *HIS3* sequence via homologous recombination. Cultures used for transformations were supplemented with 0.0033% (*w*/*v*) arginine, methionine, and tryptophan; 0.005% isoleucine, lysine, and tyrosine; 0.017% aspartate, glutamate, and leucine; 0.033% threonine; and 0.067% serine, based on prior work showing amino acid supplementation improves transformation efficiencies in yeast [64]. Individual mutations were made in the zero-copy Ty1 strain and 2-4 different high-copy Ty1 strains. Typically, 3-4 independent transformants were examined for zero- and high-copy Ty1 strains for each experiment. The absence of wild-type open reading frames in mutants was confirmed by PCR.

### 3.2. Chronological Lifespan Determination

Three different colonies were used to initiate three cultures (one colony per culture) at 5000 cells/mL in 10 mL of YPD, YPD with 30 mM hydroxyurea, YPD with 1 µg/mL of the phleomycin derivative Zeocin (Thermo Fisher Scientific, Waltham, MA, USA), YPD with 200 µg/mL G418, YPD with 30 mM hydroxyurea and 200 µg/mL G418, or SC minus uracil for each strain and condition tested. Cultures were grown on a rotator at 20–22 °C, sampled at the start of stationary phase and then sampled every 3–7 days thereafter, until viability of the populations dropped below 10%. Cell viability was determined via incubating culture aliquots in a solution of 0.25% trypan blue (*w*/*v*), 10 mM EDTA, and 0.6X phosphate-buffered saline for 40 min, followed by determining the percentage of unstained cells out of approximately 200 cells per sample through light microscopy. In our previous work [20], we observed the same relative changes in viability using trypan blue staining and spreading aging cells on fresh solid medium to allow them to form colonies, and pilot experiments for this study confirmed good correspondence (data not shown).

### 3.3. RNA Extraction for RT-PCR and RNA Sequencing

For RT-PCR, yeast cultures were initiated at 5000 cells/mL in 20 mL of YPD broth with 30 mM HU and grown in flasks with shaking at 20 °C to a density of about 1–2 × 10^7^ cells/mL. Positive control samples for *HAC1* splicing were treated with a final concentration of 1 µg/mL tunicamycin for the last 2.5 h of growth. Total RNA was prepared from 10^8^ cells per sample using the RNAqueous Total RNA Isolation Kit (Thermo Fisher Scientific, Waltham, MA, USA), according to the manufacturer’s instructions. For RNA sequencing, yeast cultures were initiated at 5000 cells/mL in 25 mL of YPD without or with 30 mM hydroxyurea and grown in flasks with shaking at 20 °C for seven days to reach stationary phase. Three biological replicates for the zero-copy Ty1 strain and three independent high-copy Ty1 strains were grown for each of the two media conditions to prepare 12 total samples. RNA was extracted from approximately 3 × 10^8^ cells per sample with the RNAqueous Total RNA Isolation Kit. RNA integrity was confirmed via agarose gel electrophoresis.

### 3.4. RT-PCR for HAC1 Splicing

Total RNA samples were treated with the TURBO DNA-free Kit (Thermo Fisher Scientific, Waltham, MA, USA) according to the manufacturer’s instructions to remove residual genomic DNA. Approximately 0.5 µg of total RNA was reverse transcribed using oligo(dT) and random hexamers using the SuperScript IV First-Strand Synthesis System (Thermo Fisher Scientific, Waltham, MA, USA). PCR was performed using equal volumes of cDNA reactions and primers to sequences flanking the *HAC1* intron (5′ to 3′): CGAACTTGGCTATCCCTACCA and CAAATGAATTCAAACCTGACTGCGCT. Mock reactions with no reverse transcriptase served as negative control templates. RNA extracted from cells treated with tunicamycin (see Section 3.3) provided a positive control for detection of spliced *HAC1* transcripts.

### 3.5. RNA Sequencing and Analysis

Total RNA was analyzed for RIN scores of >7.0 with Agilent Technologies Bioanalyzer 2100, poly(A) transcripts were purified using oligo(dT) magnetic beads and used to prepare RNA sequencing libraries for 150 bp paired-end Illumina sequencing (NovaSeq 6000) by LC Sciences (Houston, TX, USA). LC Sciences removed low-quality reads and adapter sequences using in-house perl scripts and Cutadapt before using HISAT2 to map reads against the *Saccharomyces paradoxus* reference genome available at https://www.ncbi.nlm.nih.gov/data-hub/genome/GCF_002079055.1/ accessed on 10 August 2022 (Assembly ASM207905v1). StringTie was used to assemble transcripts and determine FPKM for expression levels. Differentially expressed mRNAs were identified using the R package DESeq2. mRNAs with a false discovery rate (FDR) below 0.05 and an absolute fold change of two or greater were considered differentially expressed. Gene set enrichment analysis for Gene Ontology (GO) terms and Kyoto Encyclopedia of Genes and Genomes (KEGG) pathways were also performed by LC Sciences using annotations for *S. cerevisiae*. RNA-seq data are available at the National Center for Biotechnology Information (NCBI) Sequence Read Archive (SRA) under the BioProject accession number PRJNA1151064.

### 3.6. Plasmid Construction and Expression of Ty1 in the Zero-Copy Ty1 Strain

Construction of a control plasmid consisting of the *kanMX4* allele from the *S. cerevisiae MATalpha* deletion collection cloned into pRS426, a high-copy *URA3* plasmid, was previously described [20]. Plasmid pTy1H3*kanMX* containing the full sequence of Ty1-H3 (GenBank accession M18706.1) with *kanMX* inserted between positions 5566 and 5567 cloned into pRS426 [65] was created using the control plasmid, pJC998 (kindly provided by Joan Curcio, Wadsworth Center, Albany, NY, USA), pGTy1H3*kanMX* [20], and a PCR product amplified from pGTy1H3*kanMX*. pJC998 contains the full Ty-H3 sequence with the *his3AI[∆1]* retrotransposition indicator gene [50] inserted between the 3′ end of *TYB1* and the 3′ LTR cloned into pRS415 [65]. The PCR product was generated using a primer to positions 5240-5261 of Ty1-H3 and a primer to positions 5899-5918 of Ty1-H3 that introduced EagI and SacII sites immediately after the 3′ end of Ty1-H3. Positions 1–2173 of Ty1-H3 were cloned into the control plasmid as an Acc65I-SalI fragment from pJC998. The resulting plasmid was then a recipient for a SalI-MluI fragment from pGTy1H3*kanMX* containing Ty1-H3 positions 2173-5566 and part of the *kanMX* gene. Finally, an MluI-SacII fragment containing part of *kanMX* and positions 5567-5918 of Ty1-H3 was cloned into the plasmid. Selection for the control plasmid and pTy1H3*kanMX* was accomplished through including 200 µg/mL G418 in YPD medium or using SC medium lacking uracil (SC-ura). Plasmid retention was determined via inoculating cultures at 5000 cells/mL, growth for three days at 21 °C, spreading the same dilutions of cultures onto SC and SC-ura medium, and counting colonies after four days of growth at 30 °C. The number of colonies formed on SC-ura were expressed as a percentage of those that formed on SC medium. 

### 3.7. PCR for Differentially Expressed Gene Sequences

DNA templates were prepared via heating freshly grown colonies in a solution of 0.2 M lithium acetate and 1% (*w*/*v*) sodium dodecyl sulfate, followed by ethanol precipitation, as previously described [66]. PCR primers for differentially expressed genes are provided in Table S4. Low-copy Ty1 strains included in this analysis have three or less Ty1 insertions and have been previously described [20]. PCR products were separated using agarose gel electrophoresis and stained with ethidium bromide.

### 3.8. Statistical Analyses

All experiments were performed at least in triplicate, and specific sample sizes are noted in figure legends. Except for RNA-seq data, mean values were analyzed for significant differences using two-sided unpaired t-tests assuming equal variances. Levels of significance are indicated in figure legends.

## 4. Conclusions

**Figure 9:**
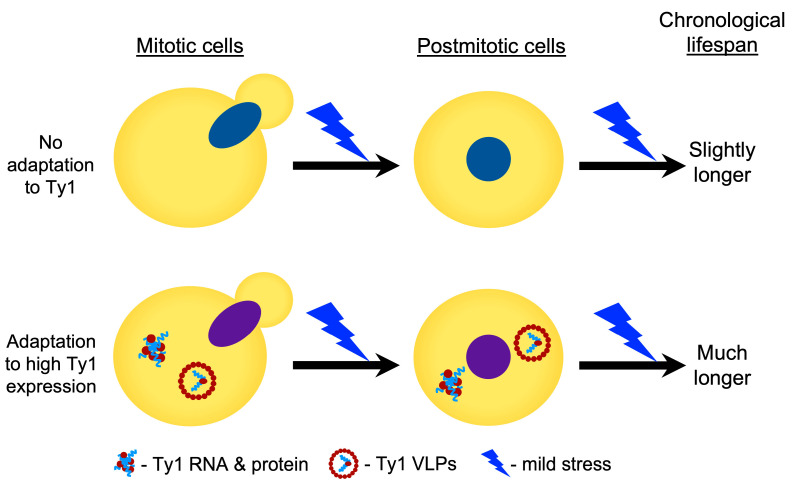
Proposed impact of Ty1 on lifespan. Cells expressing high levels of Ty1 RNA, protein, and VLPs are proposed to experience mild stress, causing an adaptive change in gene expression that results in longer lifespan upon exposure to another mild stress. The different colors for the nuclei in each row reflect the altered gene expression due to the expression of Ty1.

We propose that Ty1 expression in high-copy Ty1 strains causes a mild stress that allows cells to better respond to other mild stresses, increasing their chronological lifespan in the context of mild stress (Figure 9). This is in contrast to negative effects of retrotransposons and endogenous retroviruses on aging cells that arise due to either genetic damage or triggering innate immune responses [11,15,16,17,18]. Our prior [20] and current work indicate that the impact of Ty1 is not due to a particular pattern of Ty1 insertions affecting the expression of neighboring genes or to the level of retrotransposition, indicating that an intermediate step in Ty1 replication is likely causing cells to adapt in a manner that improves lifespan in some contexts. In consideration of a recently described increase in protein aggregation and aggregate toxicity due to Ty1 protein accumulation and VLP formation [19], we speculate that the impact of Ty1 on proteostasis is causing cells to adapt and be better prepared to age in the presence of mild stress. The study reporting the impact of Ty1 on proteostasis reported that Ty1 expression had a moderate negative effect on replicative lifespan [19]. However, distinct factors can regulate replicative and chronological lifespan [22], and the positive impact we observed for Ty1 is specific to mild stress conditions.

Overall, our work expands the association between retrotransposons and aging by showing that these transposable elements can have a positive impact in certain contexts. This is likely dependent on certain levels of retrotransposon expression that cause only a mild stress, rather than severe stress, and the level of other stresses to which cells are exposed. It will be interesting to determine whether retrotransposons or endogenous retroviruses in other organisms that contribute to cell aging can have a similar context-dependent positive impact on lifespan.

## Figures and Tables

**Table 1:** Gene set enrichment analysis (GSEA) of RNA-seq data for GO terms for high- versus zero-copy Ty1 strains grown with or without HU treatment.

High- vs. Zero-Copy Ty1 in YPD with HU			High- vs. Zero-Copy Ty1 in YPD		
GO Term	NES ^1^	FDR ^2^	GO Term	NES	FDR
Iron ion homeostasis	1.58	0.19	**S-adenosylmethionine dependent methyltransferase activity**	1.73	0.06
Glutamine metabolic process	1.61	0.20	**Ribosomal large subunit assembly**	1.61	0.08
**Establishment of mitotic sister chromatid cohesion** ^3^	1.59	0.20	**Establishment of mitotic sister chromatid cohesion**	1.61	0.09
**S-adenosylmethionine dependent methyltransferase activity**	1.51	0.22	Translational elongation	1.68	0.09
tRNA modification	1.51	0.22	Methyltransferase activity	1.62	0.10
Response to stress	1.52	0.23	Maturation of large subunit rRNA	1.63	0.10
**Carbohydrate phosphorylation**	1.53	0.24	Cytosolic large ribosomal subunit	1.65	0.11
**Ribosomal large subunit assembly**	1.51	0.24	Methylation	1.63	0.12
Microtubule nucleation	1.50	0.24	Purine nucleotide biosynthetic process	1.55	0.15
			**Carbohydrate phosphorylation**	1.56	0.15
			Cytoplasmic translation	1.55	0.16
			Large ribosomal subunit	1.57	0.16
			GPI anchor biosynthetic process	1.56	0.16
			Ran GTPase binding	1.55	0.17
			Preribosome, large subunit precursor	1.53	0.18
			Microtubule plus end binding	1.52	0.19
			Translation elongation factor activity	1.51	0.19
			One carbon metabolic process	1.51	0.19
			Condensed chromosome kinetochore	1.50	0.22
			Nucleoside metabolic process	1.49	0.23

^1^ Normalized enrichment score (overrepresentation of gene set in the list of genes, or enrichment score, divided by mean enrichment score of permutations of the dataset). ^2^ False discovery rate for significantly enriched gene sets (*q*-value). ^3^ Bold text indicates GO terms enriched for both media conditions.

**Table 2:** GSEA of RNA-seq data for KEGG pathways for high- versus zero-copy Ty1 strains grown with or without HU treatment.

High- vs. Zero-Copy Ty1 in YPD with HU			High- vs. Zero-Copy Ty1 in YPD		
Pathway	NES ^1^	FDR ^2^	Pathway	NES	FDR
**Fructose and mannose metabolism** ^3^	−1.70	0.04	**Fructose and mannose metabolism**	−1.66	0.12
Pantothenate and CoA biosynthesis	1.39	0.22	Fatty acid degradation	1.39	0.23
Arginine biosynthesis	1.52	0.23	**Pyrimidine metabolism**	1.42	0.23
Thiamine metabolism	1.48	0.23	GPI anchor biosynthesis	1.45	0.23
**Pyrimidine metabolism**	1.40	0.24	Base excision repair	1.40	0.24
Phenylalanine, tyrosine, and tryptophan biosynthesis	1.37	0.24			
Mismatch repair	1.35	0.24			
Tyrosine metabolism	1.41	0.24			

^1^ Normalized enrichment score. ^2^ False discovery rate. ^3^ Bold text indicates GO terms enriched for both media conditions.

**Table 3:** Genes overexpressed at least three-fold in high-copy Ty1 strains with HU treatment.

Gene	Description	Fold Overexpression	*q*-Value
*POP3*	Subunit of RNase MRP and nuclear RNase P	8.16	7.31 × 10^-20^
*NCS2*	Protein required for uridine thiolation of Lys(UUU) and Glu(UUC) tRNAs	5.18	4.52 × 10^-13^
*TRM9*	tRNA methyltransferase	5.10	5.55 × 10^-16^
*CDD1*	Cytidine deaminase	4.65	1.46 × 10^-16^
*FOL3*	Dihydrofolate synthetase, involved in folic acid biosynthesis	4.43	1.66 × 10^-17^
*ARG3*	Ornithine carbamoyltransferase	4.36	1.95 × 10^-17^
*AMF1*	Low affinity NH_4_^+^ transporter	4.21	2.84 × 10^-14^
*SRO77*	Protein with roles in exocytosis and cation homeostasis	4.21	3.19 × 10^-34^
*DAL1*	Allantoinase	4.20	3.67 × 10^-17^
*PMU1*	Phosphomutase	4.10	3.85 × 10^-18^
*MOG1*	Conserved nuclear protein that interacts with GTP-Gsp1p	4.09	1.31 × 10^-17^
*RHO3*	Non-essential small GTPase of the Rho/Rac family of Ras-like proteins	3.78	1.91 × 10^-14^
*THR1*	Homoserine kinase	3.62	2.51 × 10^-13^
*TAH18*	NADPH-dependent diflavin reductase	3.43	2.62 × 10^-17^
*TUB4*	Gamma-tubulin	3.37	3.20 × 10^-24^
*SPAR_D04210*	Hypothetical protein, homology to S. cerevisiae SVF1-like proteins encoded by YDR222W and YLR225C	3.29	4.94 × 10^-25^
*SWD3*	Essential subunit of the COMPASS (Set1C) complex	3.27	3.16 × 10^-15^
*YPT31*	Rab family GTPase	3.27	8.91 × 10^-18^
*ADE8*	Phosphoribosyl-glycinamide transformylase	3.24	2.24 × 10^-25^
*ACO2*	Mitochondrial aconitase isozyme	3.24	2.02 × 10^-21^
*HIF1*	Non-essential component of the HAT-B histone acetyltransferase complex	3.23	3.42 × 10^-13^
*RIB3*	3,4-dihydroxy-2-butanone-4-phosphate synthase (DHBP synthase)	3.21	5.63 × 10^-30^
*NCS6*	Protein required for uridine thiolation of Gln, Lys, and Glu tRNAs	3.10	5.00 × 10^-16^
*PRS1*	5-phospho-ribosyl-1(alpha)-pyrophosphate synthetase	3.02	1.06 × 10^-32^

## Data Availability

RNA-seq data discussed in this article are available at the National Center for Biotechnology Information (NCBI) Sequence Read Archive (SRA) under the BioProject accession number PRJNA1151064. Additional raw data not presented in the study supporting the conclusions of the article will be made available by the authors on request.

## Supplementary Materials

The following supporting information can be downloaded at: https://www.mdpi.com/article/10.3390/ijms251910593/s1.
