# Supplementary material for: Lifespan Extension by Retrotransposons under Conditions of Mild Stress Requires Genes Involved in tRNA Modifications and Nucleotide Metabolism
Source: Int J Mol Sci. 2024 Oct 1;25(19):10593. doi: 10.3390/ijms251910593 (PMC11477299; doi:10.3390/ijms251910593)
Supplement: Supplementary file 1 [file ijms-25-10593-s001.zip › Figure S1.pdf]

```
  1 ------ATGACTGATTTTGAACTAACTAGTAATTCGCAATCGAACTTGGC     44
          ||||||||||||||||||||||||||||||||||||||||||||
  1 ATGGAAATGACTGATTTTGAACTAACTAGTAATTCGCAATCGAACTTGGC     50

 45 TATCCCTACCAACTTCAAGTCGACTCTGCCCCCAAGGAAAAGAGCGAAGA     94
    ||||||||||||||||||||||||||||||.||||||||||||||.||||
 51 TATCCCTACCAACTTCAAGTCGACTCTGCCTCCAAGGAAAAGAGCCAAGA    100

 95 CAAAAGAGGAAAAGGAACAGCGAAGGATCGAGCGAATTTTGAGAAACAGA    144
    ||||||||||||||||||||||||||||||||||.|||||||||||||||
101 CAAAAGAGGAAAAGGAACAGCGAAGGATCGAGCGTATTTTGAGAAACAGA    150

145 AGAGCTGCTCACCAGAGTAGAGAGAAAAAGAGACTACATCTGCAATACCT    194
    |||||||||||||||||.|||||||||||.||||||||||||||.||.||
151 AGAGCTGCTCACCAGAGCAGAGAGAAAAAAAGACTACATCTGCAGTATCT    200

195 CGAGAGGAAATGTTCTCTTTTGGAAAATTTGCTGAACAGTGTCAACCTTG    244
    ||||||.|||||||||||||||||||||||.||||||||.||||||||||
201 CGAGAGAAAATGTTCTCTTTTGGAAAATTTACTGAACAGCGTCAACCTTG    250

245 AAAAATTGGCTGACCACGAGGACGTGTTGACTTGTGGCCACGATGCTTTT    294
    |||||.|||||||||||||.||||.|||||||||..|||||||.||||||
251 AAAAACTGGCTGACCACGAAGACGCGTTGACTTGCAGCCACGACGCTTTT    300

295 GTTGCTTCTCTTGACGAGTATAGGGATTTCCAGAGCACGAGAGACGCCTC    344
    ||||||||||||||||||||.||||||||||||||||||||.|.|||.||
301 GTTGCTTCTCTTGACGAGTACAGGGATTTCCAGAGCACGAGGGGCGCTTC    350

345 ACTGGACGCCAGGGCCAGTTCGCACTCATCGTCTGATACGTTCACACCTT    394
    |||||||.|||||||||||||||||||.||||||||||||||||||||||
351 ACTGGACACCAGGGCCAGTTCGCACTCGTCGTCTGATACGTTCACACCTT    400

395 CACCTTTGAACTGTACAATGGAGCCTGCAACTTTGTCGCCCAAGAGTATG    444
    |||||.||||||||||||||||||||||.|||||||||||||||||||||
401 CACCTCTGAACTGTACAATGGAGCCTGCGACTTTGTCGCCCAAGAGTATG    450

445 CGTGATTCTTCGTCGGACCAAGAGACTTCATGGGAACTGCAGATGTTTAA    494
    ||.|||||..|||||||||||||||||||||||||.||||||||||||||
451 CGCGATTCCGCGTCGGACCAAGAGACTTCATGGGAGCTGCAGATGTTTAA    500

495 GACGGAAAATATACCAGAATCCACGACGCTGCCTGCCGTAGACAACAACA    544
    ||||||||||.|||||||.||.||||||||.|||||||||||||||||||
501 GACGGAAAATGTACCAGAGTCGACGACGCTACCTGCCGTAGACAACAACA    550

545 ATTTGTTTGATGCGGTGGCTTCGCCGTTGGCAGACCCGCTCTGCGACGAT    594
    |||||||||||||||||||.|||||||||||||||||.||||||||||||
551 ATTTGTTTGATGCGGTGGCCTCGCCGTTGGCAGACCCACTCTGCGACGAT    600

595 ATAGCGGGAAACAGTCTACCCTTTGACAATTCAATTGATCTTGACAATTG    644
    ||||||||||||||||||||||||||||||||||||||||||||||||||
601 ATAGCGGGAAACAGTCTACCCTTTGACAATTCAATTGATCTTGACAATTG    650

645 GCGTAATCCAGCCGTGATTACGATGACCAGGAAACTACAGTGAACAAGAA    694
    ||||||||||||||||||||||||||||||||||||||||||||||||||
651 GCGTAATCCAGCCGTGATTACGATGACCAGGAAACTACAGTGAACAAGAA    700

695 GACCAGCCCCAATTTTTACTTTCTGCAT------TTTTTTTTTTTTTTTA    738
    .||.|||||||..||||.||||||||.|      ||||||||||||||||
701 CACTAGCCCCAGCTTTTGCTTTCTGCTTTTTTTCTTTTTTTTTTTTTTTA    750

739 GTCGTGGTTCTCTAAAGGGGGAGGAGCCGGTTAAAGTACCTTCACAAAAG    788
    |||||||||||||.|.||||||||||||||||||||||||||  ||||||
751 GTCGTGGTTCTCTGATGGGGGAGGAGCCGGTTAAAGTACCTT--CAAAAG    798

789 CAGAATGCAAGAGTATTGGGAGCAGTTTGTTTTTT--TTTCATGCTAGTT    836
    |||||||||.|..||||||.|||  |||.||||||  |||.||||||| |
799 CAGAATGCAGGGTTATTGGAAGC--TTTCTTTTTTTCTTTTATGCTAG-T    845

837 TTTTCCTGAACAAAAAGAGCC-TTCTTTCCTTATTGTTAGGGAATAGGTG    885
    ||||||||||||||.|||||| ||||||.||||||..||.|.|||.|..|
846 TTTTCCTGAACAAATAGAGCCATTCTTTTCTTATTACTAAGAAATGGACG    895

886 GCTTGCTTGTACTGTCCGAAGCGCAGTCAGGTTTGAATTCATTTGAATTA    935
    |||||||||||||||||||||||||||||||||||||||||||||||||.
896 GCTTGCTTGTACTGTCCGAAGCGCAGTCAGGTTTGAATTCATTTGAATTG    945

936 AACGATTTCTTTATCACTTCGTGA    959
    ||.||||||||.||||||||.|||
946 AATGATTTCTTCATCACTTCATGA    969
```

Figure S1. Alignment of *S. paradoxus* (upper) and *S. cerevisiae* (lower) *HAC1* sequences. S. paradoxus sequence is positions 95,579 to 96,537 in GenBank accession NC_047492. S. cerevisiae sequence is positions 75,179 to 76147 in GenBank accession NC_001138. Region boxed in blue indicates the intron sequence present in *S. cerevisiae*. Sequences in yellow indicate positions of PCR primers. Alignment was performed using the EMBOSS Needle pairwise sequence alignment tool (Madeira et al., 2024, reference 36 in main text).
