# Supplementary material for: Lifespan Extension by Retrotransposons under Conditions of Mild Stress Requires Genes Involved in tRNA Modifications and Nucleotide Metabolism
Source: Int J Mol Sci. 2024 Oct 1;25(19):10593. doi: 10.3390/ijms251910593 (PMC11477299; doi:10.3390/ijms251910593)
Supplement: Supplementary file 1 [file ijms-25-10593-s001.zip › Table S4.pdf]

Table S4. Primers used to analyze differentially expressed gene sequences

Gene name	Sequence 5'-3'	Note
AMF1	TGGCATTGCTTGGCACTTGTCC	upstream of coding region
AMF1	GCAAGGACCAAAGGGCGTACC	coding region
ARG3	GGTCAAAACCAACCTCACTTGAG	upstream of coding region
ARG3	CGTTGATGCTGTGGTTGACATGG	coding region
BSC5	AGCGAGAGCAAGTGAGGCTTG	upstream of coding region
BSC5	GTATGGGAATCGCTCGCCTTG	coding region
BSC5	GCAAACAGGCTTGGAAGGATCG	coding region
BSC5	CGTGAGCTTTCGCTACCACGT	downstream of coding region
CDD1	GACACCCGGTATTACTCGAGTACG	upstream of coding region
CDD1	CCATTCGGAAATTGGCGAGCAGC	coding region
CIS3	GAGATGGATCCGGTCTGGTAGG	upstream of coding region
CIS3	GAGTGGCTTGGACTTGACCGT	coding region
CTP1	ACTAGCCTTGCAAGAAGACAGC	upstream of coding region
CTP1	GCTCTCCAGTTTCACGGTCTC	coding region
DAL1	GCCTATCAATGCCATCACTTCC	upstream of coding region
DAL1	GGATTGTTGCCGTAGAGACTC	coding region
DAL7	CGCTGTTCTGCTGCATGACGA	upstream of coding region
DAL7	GTTGATCAAGCCAGGAGCAGG	coding region
DSE4	TGCGCTGTAAGCTGCGCATCTG	upstream of coding region
DSE4	GGAACTGTCATAACCACCGTAGC	coding region
DSE4	TCACCAGCATTTGTGTACCCT	coding region
DSE4	GCCCATGTGCGACTCATTCCGT	downstream of coding region
DSE4	AGAGATGTCGCCAATCCATCTG	coding region
ENB1	TCTCCAGAGATCACGCCAGAAC	upstream of coding region
ENB1	CGAAATCTGCCCACCAAGTCC	coding region
FOL3	GGGCCCTGCTGTCGATGTAGG	upstream of coding region
FOL3	CCGCACACAACGATTGGTCGT	coding region
HXT17	CACCGACGTTGATTTCATCAGC	coding region
HXT17	GAACAGTGAAACCTCCTTGCCA	upstream of coding region
IPI3	GCAATTCCATCATGCTCAGTCCC	upstream of coding region
IPI3	CAGAAGCTACCGTCCCTGAGG	coding region
KAP114	GTCTTTCCACTATCGTCTGCCC	upstream of coding region
KAP114	CCTCTCTAGTGCGTTTATCAGCTG	coding region
NCS2	CAGCCTTGTCGTAACTTTCAGG	upstream of coding region
NCS2	CGAGTACCGAGATAGGGATGG	coding region
NSE5	GATCTACTTCCGTCGGAAGTGG	upstream of coding region
NSE5	GCTCAATGTTTCCCTGGACACG	coding region
OAC1	CCCCCGTCACATGTGCTTCCA	upstream of coding region
OAC1	GGTCCCTTGTACAAGTCACCC	coding region

PDR18	CGGTGAAGAACATGTCCTGCT	upstream of coding region
PDR18	GAACCATGGGTGCATTGAAGG	coding region
PDR18	CAGGCCAACTCTATTGCTGGT	coding region
PDR18	GGACTATATGTTCACGCCCATG	downstream of coding region
PDR18	GCAAACCCAGACGCGACATCAG	coding region
PMU1	CGTATCATGGACTGAGAGCCAC	upstream of coding region
PMU1	CCTTGACCATGTCTAGCCAAG	coding region
POP3	GGGTCCGCGGTAGAGGCAGC	upstream of coding region
POP3	CCTGTCGTCGCACCGCAGCAAC	coding region
RPL30	GATCTTGGAGTCACCACGGAC	upstream of coding region
RPL30	GTAGTAGACCTTGGTCTTGGAC	coding region
RPL5	TGGCATATGCAGCGGTACCAC	upstream of coding region
RPL5	ACCAGTAGCGTAAGCAGCAGC	coding region
RPP0	GCGCTGGCCTTACTGTAGGCA	upstream of coding region
RPP0	AGTGTTAACGGCTCTAACCCAG	coding region
RPP2B	CTTACCAACACCCGGTAACGC	upstream of coding region
RPP2B	CGACGGCCTTGATGTCGGCG	coding region
SLA2	CTCGTCACTCAGTTTAGCATCC	upstream of coding region
SLA2	GTCTCCTCGACGGAACACGCC	coding region
SPAR_I00020	GCCCTTGCCCTTATCTCCAGAG	upstream of coding region
SPAR_I00020	TCAGACAGGACCCTGTTGACG	coding region
SPAR_N03660	AAGCTTCGGCTCCTGCAACAC	coding region
SPAR_N03670	CACGTAGTTCCGCCCACAAGC	coding region
SPAR_N03670	TGGCATACCTAATTGCCTGGCT	coding region
SPAR_N03670	GGCTCTAACTATACCCATCCCTG	coding region
SPAR_N03670	GCATCGAAACTTATCCACACTGC	coding region
SPAR_N03710	ATGCCAACCGTATTGCGAAGG	coding region
SPAR_N03710	GCAGCTGGAGAGGAATGGGCA	downstream of coding region
SPAR_N03710	GCGCAATGGTGGCTTGAAACC	coding region
SPAR_N03730	ATGGCGATGGTGAGGGTGGTG	downstream of coding region
SPAR_N03730	GATGCAGGAGCACCATTTGGAG	coding region
SPAR_N03740	GTCCCATGTGACTTTACCCTCG	upstream of coding region
SPAR_N03740	TCTCTCAGTAGGAGCACCTTGC	coding region
SPAR_P02760	CGCTTGCAAGATGCCCAACCAG	upstream of coding region
SPAR_P02760	CAGGGTGGAAGATGCACATCC	coding region
SRO77	ACTCATGCTTCCAGAGCCACT	upstream of coding region
SRO77	CTCTCAAGACCGATCAGCATCC	coding region
THI4	CGGTATTGTTGTTGGCGCAAAC	upstream of coding region
THI4	GCCATAGCGCCGAAAGTTGGACC	coding region
TRM9	GGAGCCGCTGCACATGGTCTG	upstream of coding region
TRM9	CTCTTCTGGGCCACCACCCACC	coding region
